# Asylum or Hospital?

**Published:** 1892-08-20

**Authors:** 


The Hospital

An Institutional Journal of

Science, Medicine, Nursing, and Philanthropy.

*For Hospitals, Asylums, Infirmaries, Dispensaries, Medical Practitioners, Students, Nurses, and the Charitable Public.*

Vol. XII.—No. 308.] [Aug. 20, 1892.

### Asylum or Hospital?

Two circumstances recur to the mind of the writer which illustrate two clearly recognised phases in the history of the medical treatment of the insane. More than thirty years ago, in a certain small town in the north of England, a small boy used to pass on his way to school the house of the parish constable. That was before the days of the rural police. There was often to be seen standing at a bedroom window in the constable's house a form fearful to small boys: it was that of a mad woman, who went by the name of Nelly Moore. Nelly was often teased by passers-by, even by those who were beyond the age of childhood. When teased she would speedily work herself into a rage terrible to behold; and no one doubted that if she could have forced her body through the bedroom window she would have jumped down into the street and torn her tormentors to pieces. But that was impossible, for she had a stout iron ring or chain round her body, and to this was attached an iron chain, by which she was securely fastened to one of the solid posts of an old-fashioned four-post bed. Moreover, she was handcuffed. As poor Nelly Moore was treated in that northern town, so were thousands more in all parts of the civilised world thirty years ago. They were looked upon as savage and dangerous wild beasts, and were caged or chained up accordingly.

About a score of years ago the same boy, then a man, was attending lunacy lectures with a senior class of medical students at a well-known lunatic asylum. On one occasion the lecturer, after showing several patients and explaining the peculiarities of each case, introduced a middle-aged lady of intelligent and benevolent aspect. "This lady," said the doctor, "is now well, and will return home to-morrow to take charge of her family, which she is quite competent to do. I shall say nothing about her: she will tell you her own story." The lady then, in the easiest and most natural way, told the assembled students and practitioners the story of her lunacy. "This is the third time I have been here," she said, "and this time I came of my own accord. Dr. ——," she added, looking gratefully at the medical superintendent, "has three times saved me from suicide." The story of the lady was that she had gradually developed suicidal mania at the age of a little over forty. There was no hereditary tendency in her family, and her mania had been witnessed with wonder and blamed as a fault, until a determined attempt at suicide had opened the eyes of her friends. Then, for the first time, she was put under restraint. The horror, she said, which she experienced when she first realised that she was actually an inmate of a lunatic asylum was overwhelming and indescribable. In the course of two or three years she completely recovered, and resumed her duties at home. A relapse took place, which, when her friends recognised, they knew would be best treated at the asylum, and accordingly, without much opposition on her part, she was a second time placed under restraint. She immediately began to recover, and was restored to health in less than half the time which had been required for the successful treatment of her first attack. After being at home for some few years, she felt the early symptoms of a third attack coming on; and this time, instead of waiting for her friends to act, she herself insisted upon being taken to the asylum as to a place of rest and security. A few weeks there were sufficient to restore her to perfect health.

These two cases show quite clearly the difference between rational and irrational, right and wrong methods of treating the insane. Nelly Moore, who was presumably a pauper lunatic, was regarded as a dangerous wild beast, and was chained and imprisoned accordingly. The lady, who recovered, was treated as a human being suffering from a distressing illness. The utmost kindness was shown her, and every spark of reason which she had remaining, and she had many, was guarded with care and fanned into a flame. The result was perfect cure. To her the asylum was no prison, and chains and handcuffs were unknown. It was a hospital, and a hospital only; and she herself came at last to recognise this fact so completely that the institution not only had no terrors for her, but had secured a lodgment in her mind as the only place of safety and mental repose when she was threatened by a return of her enemy. "Take me back to the hospital, and to Dr. ——," was then her only wish.

The point of these two illustrations is that a lunatic asylum is really and truly a hospital and nothing else. It ought to be conducted, in every department, entirely as a hospital. A very large proportion of lunatics are suffering from a disease which is curable. Very many of those who cannot be brought back to perfect reason can be so taught, trained, and disciplined that they shall have developed in them a kind of new reason, with a new conscience and a new will. The lunatics that are absolutely hopeless are exceedingly few in number. All this is matter of common knowledge to lunacy specialists of average intelligence. What we complain of is that this common knowledge is, to a large extent, unappreciated by the general public, and, what is worse, is practised only on a very limited scale at ordinary lunatic asylums. By the public, asylums are still regarded as cages or prisons for the immuring of creatures that, like untamed savage beasts, are intractable and dangerous. Those who have charge of lunatics in the capacity of nurses and attendants very often share the ignorance and irrationality of the uninstructed public. The practical outcome of this is that the average lunatic is still regarded rather as a prisoner than as a patient, and the asylum as a house of detention for the dangerous rather than as a hospital for the cure of the sick.

Specialists write to us from asylums complaining of the ignorance and incapacity of nurses and attendants, and of the dull indifference of medical officers, which sometimes gives place to a dogged resistance of every attempt at reform. This knowledge comes upon many of us with a shock of surprise. Knowing, as we do, that science has long ago shown lunacy to be a definite symptom and result of brain disease, we cannot but be painfully disappointed at the slowness with which these true and worthy expositions of science make their way among those who are responsible for the care and cure of lunatics. To take an example: in some asylums it seems to be still the rule to treat those who are mentally diseased like flocks of sheep or herds of dumb-driven cattle. They dine together in scores at long, narrow tables, as sheep in the field eat from their narrow troughs, or sometimes, it would appear, even like pigs in the sty, rushing greedily at their food to see which of them can gulp it down with the rudest haste. It is well known that order, method, and decency are among the primest of all instruments for the treatment and cure of the insane. Reason has to be redeveloped from its rudiments, and this is to be done, as in the training of children, by insistence upon hourly obedience to rules, rather than by the mere teaching of general principles. It is the concrete which the diseased and enfeebled mind can, and will, understand and lay hold of. The merely abstract is difficult, painful, impossible.

There are few people who recognise that lunacy is for the most part a form of brain exhaustion. The brain of the lunatic, or a portion of it, is feeble from the long-continued diminution of its blood supply; or the whole brain, or a portion of it, has been called upon by the conflict of life to make exertions which it was not able to continuously sustain, and so like the heart of the over-trained athlete, it has given way. This very illustration helps us to see on what principles lunacy is to be treated, and with what patience those principles are to be carried out. The heart of the athlete can probably never be made so elastic, so resilient, so firm and so enduring as it was before. But by patience and intelligent management it may be made comfortable and serviceable up to a certain point. Similarly the brain of the lunatic will, in a very large number of instances, if managed with practical reason, come again to play the part of moderate rationality.

It seems hard, peculiarly hard, that lunatics, at this advanced stage of medical science, should have to plead at the bar of the concrete representatives of that science for its rightful application in their own case. Every medical superintendent of a lunatic asylum, and every nurse even, will tell us in words that the asylum is a hospital, not a mere house of detention. But in practice many of those very superintendents and nurses make their institutions houses of detention, prisons, and nothing else. When will the day dawn that will emancipate all mental patients from the thrall of the jailer, and place them under the control and care of the trained nurse, and of the physician who works for cures?

